# Diffusion–Model–Driven Discovery of Ferroelectrics for Photocurrent Applications

**DOI:** 10.1002/advs.202522108

**Published:** 2026-03-28

**Authors:** Byung Chul Yeo, Hyun‐Jae Lee, Sungwoo Kang, Jung‐Hoon Lee

**Affiliations:** ^1^ Department of Energy Resources Engineering Pukyong National University Busan Republic of Korea; ^2^ Computational Science Research Center Korea Institute of Science and Technology (KIST) Seoul Republic of Korea; ^3^ Division of Nanoscience and Technology KIST School University of Science and Technology (UST) Seoul Republic of Korea; ^4^ KU‐KIST Graduate School of Converging Science and Technology Korea University Seoul Republic of Korea

**Keywords:** diffusion model, ferroelectric materials, generative AI, machine‐learning interatomic potential, polarization

## Abstract

Ferroelectric materials are vital for next‐generation memory and photovoltaic technologies, yet their discovery is limited to a few known prototypes. Here, we present a design framework that integrates diffusion–model–based crystal generation with multi‐fidelity screening to the discovery of ferroelectrics. Using MatterGen, we generated 12 800 candidate structures and applied a pipeline combining diverse machine‐learning tools and density functional theory calculations. This process revealed two promising candidates, Ca_3_P_2_ and LiCdP, both insulating and switchable. The polarization value of Ca_3_P_2_ is 8.9 µC cm^−2^ while that of LiCdP reaches as high as 144.1 µC cm^−2^ which is slightly higher than that of Sc‐doped AlN, one of the highest‐polarization ferroelectrics reported to date. Notably, the low‐temperature crystal structure of Ca_3_P_2_ has not been previously identified, and our study reveals a plausible candidate for this phase. In addition, HSE06 calculations yield bandgaps of 1.58 and 1.13 eV for Ca_3_P_2_ and LiCdP, respectively, suggesting strong potential for photocurrent applications. These findings establish new promising candidates for the ferroelectric family and demonstrate the power of generative models to uncover novel functional materials.

## Graphical abstract

We developed a diffusion model–based generative AI and high‐throughput screening framework that accelerates the discovery of photovoltaic ferroelectrics. By coupling AI driven crystal generation with machine learning and DFT screening, we identified Ca_3_P_2_ and LiCdP as new ferroelectric materials exhibiting strong polarization, feasible switching barriers, and ideal bandgaps, offering valuable insights for both experimental and computational materials scientists.

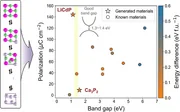

## 1 Introduction

Ferroelectric materials have an electric polarization that can be switched with external electric fields. These properties have enabled widespread applications in nonvolatile ferroelectric random‐access memories [1], piezoelectric sensors [2], energy harvesting devices [3], and optoelectronic applications [4]. In particular, ferroelectrics exhibit unique photocurrent responses arising from their non‐centrosymmetric crystal structures and switchable polarization [5, 6, 7, 8]. Previous studies have demonstrated their potential in ferroelectric photovoltaics, where above‐bandgap photovoltages and polarization‐dependent photocurrents can be exploited [9, 10, 11, 12].

Therefore, the discovery of new ferroelectric materials with enhanced photocurrent properties is critical for designing next‐generation switchable devices that integrate ferroelectric, electronic, and optical functionalities. However, despite significant efforts in experimental synthesis and density functional theory (DFT) calculations, the known chemical space of ferroelectrics remains relatively narrow, largely confined to perovskites, layered oxides, and a few organic‐inorganic hybrids [13, 14]. This scarcity presents a fundamental challenge, as ferroelectrics are essential for next‐generation devices, yet there are few established libraries to guide the discovery of novel materials.

Traditional materials discovery approaches often rely on human intuition, trial‐and‐error synthesis, or brute‐force high‐throughput DFT screening within known structural families [15]. While successful to some extent, these methods are inherently constrained by human bias and computational cost. In recent years, generative models have emerged as powerful tools for data‐driven materials design [16, 17, 18, 19, 20, 21]. These models can learn the underlying statistical distribution of sampled crystal structures and generate novel structures that reflect this distribution while exploring new compositions. Thus, such models offer the potential to systematically explore the vast and under‐investigated chemical spaces that are likely to host undiscovered ferroelectrics.

Among various architectures, diffusion models have shown excellent performance in image and molecular generations, outperforming earlier approaches such as variational autoencoders and generative adversarial networks [22, 23, 24]. Recently, diffusion models have also been introduced in materials science, showing promising results for inorganic crystals. Among them, MatterGen and DiffCSP have received considerable attention [16, 19]. Both are diffusion models trained on hundreds of thousands of crystals and are capable of generating chemically plausible crystal structures with high diversity and reliability. To harness this potential for materials discovery, we implement a rigorous screening workflow to identify promising ferroelectric candidates from the generated structures for optoelectronic applications.

In this work, we have developed a generative artificial intelligent (AI)–driven workflow to discover novel ferroelectric materials by integrating a multi‐fidelity screening pipeline. While diffusion models can produce a wide range of stable crystal structures, evaluating their suitability as functional materials requires systematic downstream validation. The generated structures were assessed across multiple criteria, considering thermodynamic stability, ferroelectric photovoltaic characteristics, and metastability using several machine‐learning (ML) tools and DFT calculations. This workflow bridges generative crystal design with physical validation, enabling the efficient identification of previously unknown, yet realistically synthesizable ferroelectric materials. The final candidates were further analyzed by calculating detailed electronic and phonon band structures.

## 2 Results and Discussion

### 2.1 Screening of Materials Generated From Diffusion Model

**FIGURE 1:**
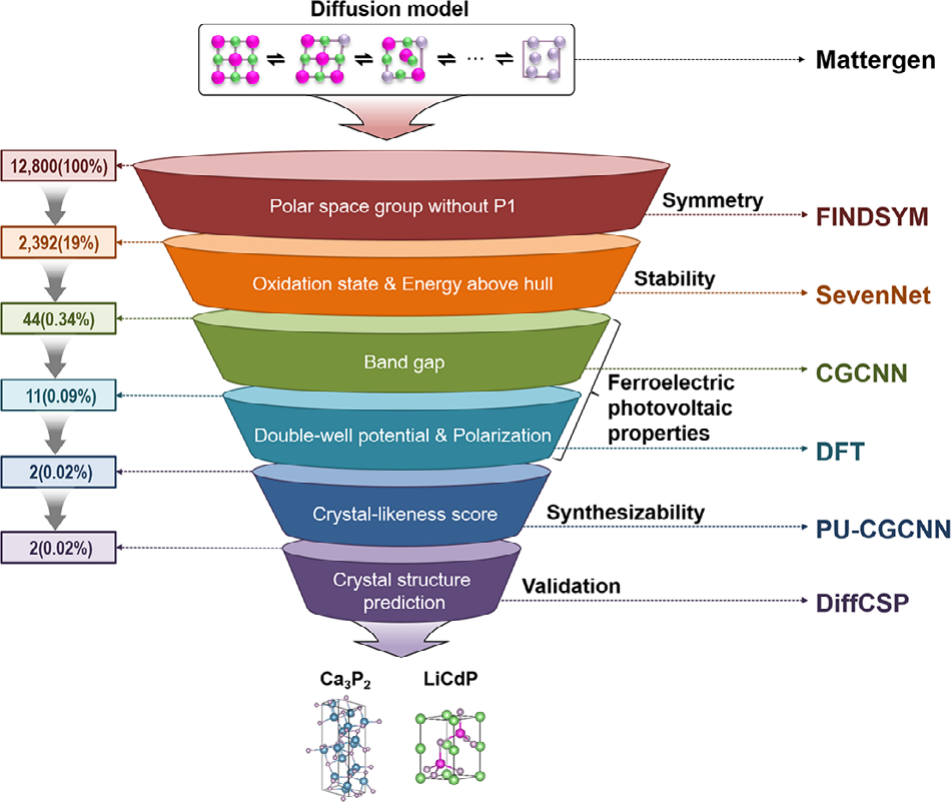
Schematic illustration of the sequential filtering pipeline applied to generated crystal structures via diffusion models. The first stage (red) applies symmetry filtering by polar space groups. The second stage (orange) checks stability through the oxidation state and energy above the hull. The third stage (green) screens the bandgap, a key ferroelectric photovoltaic property. The fourth stage (light blue) screens the double‐well potential and polarization, two key ferroelectric photovoltaic properties. The fifth stage (dark blue) assesses synthesizability with a crystal‐likeness score. The final stage (purple) validates candidates through the crystal structure prediction. This process ultimately identifies Ca_3_P_2_ and LiCdP as promising ferroelectric candidates for photocurrent applications.

We applied our screening workflow to a set of crystal structures generated *de novo* by MatterGen [16]. The initial dataset was constructed by generating a finite pool of candidate structures under composition and symmetry conditions relevant to our target material class. From the generative pool, successive filters reduced 12,800 initial structures to a small set of chemically valid and previously unreported candidates. As shown in Figure 1, only a fraction survived the symmetry, stability, synthesizability, and ferroelectric photovoltaic properties constraints, with further screening and validation ultimately yielding two promising ferroelectric candidates.

The ordering of the screening steps shown in Figure 1 was deliberately designed to maximize computational efficiency while preserving physical relevance. Although stability‐related filters could, in principle, be applied prior to symmetry analysis, we chose to begin with symmetry‐based filtering because it is computationally inexpensive and immediately excludes structures that cannot exhibit ferroelectricity. This initial step reduced the generative pool from 12,800 to 2,392 candidates (≈19%), substantially lowering the computational cost of subsequent stability and property evaluations. By enforcing symmetry constraints upfront, all downstream screening steps are applied only to candidates that are intrinsically capable of exhibiting ferroelectric behavior, enabling a targeted discovery strategy rather than a brute‐force, stability‐first screening.

**FIGURE 2:**
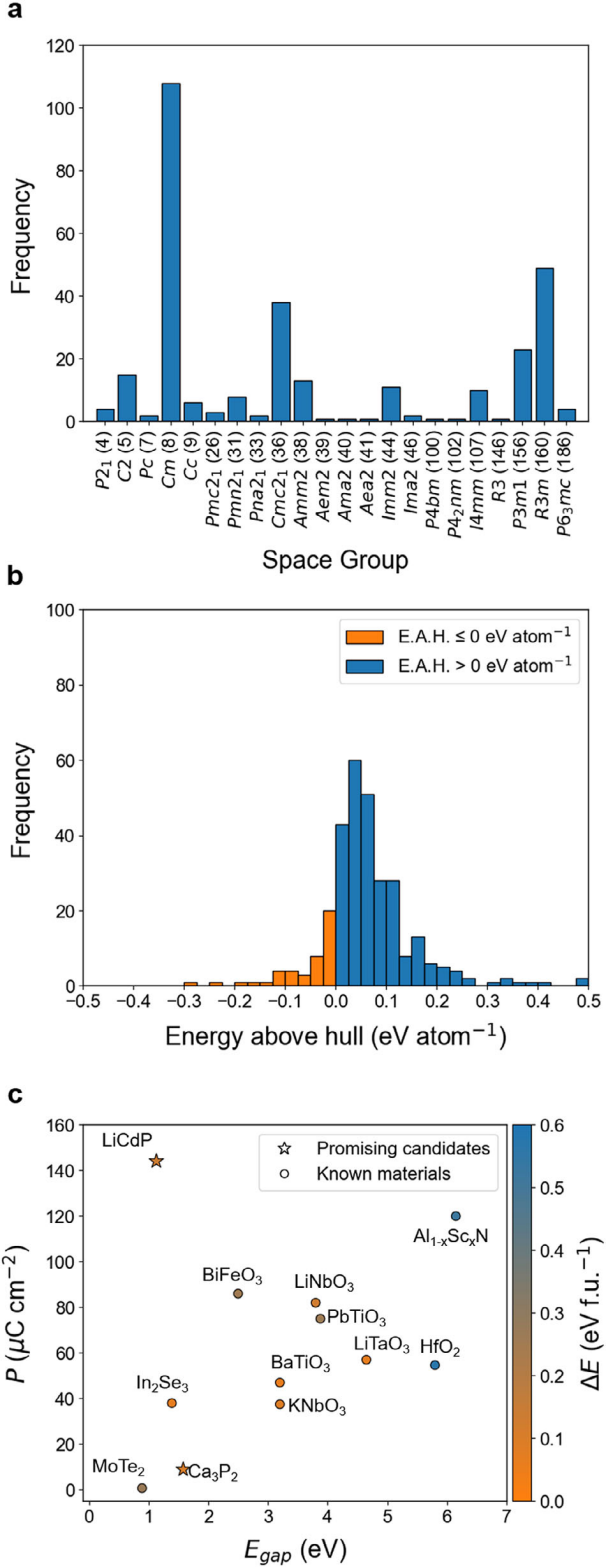
Screening results for symmetry, stability, and ferroelectric properties. a) Histogram of polar space groups (excluding P1) showing the structural diversity retained after the initial symmetry filter. b) Distribution of the energy above hull for screened structures with bars colored orange for compounds within 0 meV atom^−1^ and blue for those above this threshold. c) Scatter plot of bandgap (eV) versus spontaneous polarization (µC cm^−2^); marker color indicates the double‐well energy difference (eV f.u.^−1^). Stars denote the two promising candidates (Ca_3_P_2_ and LiCdP) identified in this work, and circles denote known ferroelectrics (BaTiO_3_ [25, 26], PbTiO_3_ [25, 27], KNbO_3_ [25, 28], LiNbO_3_ [29, 30], LiTaO_3_ [29, 31], HfO_2_ [32, 33], MoTe_2_ [34, 35], In_2_Se_3_ [36], Al_1‐x_Sc_x_N [37, 38], and BiFeO_3_ [39, 40]). For Ca_3_P_2_ and LiCdP, the polarization and double‐well energy difference were obtained from PBE‐level calculations while the bandgap values were obtained from HSE06 calculations. For the known ferroelectrics, the polarization and double‐well energy difference were also obtained from PBE‐level calculations, whereas the bandgap values correspond to experimental values.

The workflow begins with symmetry analysis using FINDSYM [41], where only structures belonging to polar space groups (except P1), as defined from a survey of polar materials in the Materials Project database [42], were retained (Table S1 for details). This filtering alone eliminated nearly four‐fifths of the generative pool, leaving 2,392 candidates which are potentially switchable. The structural diversity retained after this initial filtering is summarized in Figure 2a, which highlights the distribution of polar space groups across the surviving candidates.

In addition, because the diffusion model is trained on the Alex‐MP‐20 dataset, which is restricted to structures with relatively small unit cells and near‐equilibrium thermodynamic stability, the generative process may preferentially sample chemistries and coordination environments that are well represented in existing databases. As a result, compounds requiring larger or more complex unit cells, more metastable phases at higher energies above the convex hull, and uncommon coordination motifs may be sampled less frequently or missed altogether. These considerations indicate that the present workflow is intended as a targeted discovery strategy for symmetry‐driven ferroelectrics, rather than a comprehensive exploration of all polar or correlation‐dominated materials.

Following symmetry‐based filtering, the remaining candidates were further screened by oxidation states and thermal stabilities. The oxidation‐state analysis served to exclude chemically implausible valence configurations, ensuring that only charge‐balanced compositions were considered. Specifically, we required an integer charge neutral oxidation state assignment using only the allowed values shown above each element in Figure S1 [43]. Compositions that could not be balanced or that required oxidation numbers not listed in Figure S1 were discarded. The thermodynamic stability of each candidate was evaluated by its energy above convex hull, referenced to the Materials Project database. Structural optimizations and energy evaluations were performed using SevenNet‐0 [44], a universal machine‐learned interatomic potential (MLIP) with an equivariant graph architecture that extrapolates well to untrained compositions [45]. We selected materials with zero or negative hull energies, indicating their thermodynamic stability against decomposition into reference phases. Applying these criteria reduced the pool to 44 structures (Figure 2b), as summarized in Table S2.

To evaluate the suitability of photovoltaic ferroelectrics, we considered three key descriptors: a finite bandgap, the presence of a double‐well potential, and a sizable spontaneous polarization. As the first step within this stage, the bandgaps were rapidly predicted using the crystal graph convolutional neural networks (CGCNN) model [46]. Candidates predicted to be metallic or with bandgaps below 0.5 eV or above 2.0 eV were discarded. This criterion was motivated by the known optimum for single‐junction solar energy conversion, which typically lies around 1.4–2.3 eV [47], while intentionally using a slightly broader window to account for systematic and model‐related uncertainties. In particular, the Perdew–Burke–Ernzerhof (PBE) exchange–correlation functional is known to underestimate bandgaps [48], and our CGCNN model was trained on PBE bandgap data from the Materials Project, which can introduce additional prediction errors. Applying this filter left eleven candidates (Table S3).

Then, nonpolar reference structures corresponding to each polar candidate were generated automatically using the PSEUDO code from the Bilbao Crystallographic Server (BCS) [49]. Among the high‐symmetry phases suggested, only nonpolar ones were retained and ranked according to their maximum atomic displacement (MAD) relative to the polar phase. The nonpolar structure with the smallest MAD was selected as the reference, providing a consistent and physically meaningful benchmark for double‐well potential and polarization calculations. This procedure excluded cases where the PSEUDO code yielded nonphysical or chemically implausible references, while retaining only symmetry‐related nonpolar counterparts with minimal expected atomic displacements, thereby narrowing the set to a small number of viable candidates. We further performed DFT calculations to confirm the bandgaps (*E_gap_*) and energy differences (Δ*E*) between polar and nonpolar structures for these candidates shown in Table S3. By doing this, metallic and non‐switchable structures were ruled out.

It is worth noting that our MLIP shows accuracy in the total‐energy prediction. To assess the reliability of the MLIP–predicted energies used in the convex hull screening, we compared total energies calculated with SevenNet‐0 to DFT total energies for the eleven candidates listed in Table S3. As shown in Table S4, the absolute deviations in total energy, |*E*_*tot*,*DFT*_ − *E*_*tot*,*SevenNet* − 0_|, are all below 60 meV per atom. The resulting root‐mean‐square error (RMSE) is 26.5 meV per atom, comparable to typical uncertainties arising from the choice of exchange–correlation functional and *k*‐point convergence in DFT calculations [50, 51, 52]. These results indicate that SevenNet‐0 provides sufficiently accurate energetic ordering for high‐throughput convex hull screening. To further mitigate the risk of false positives near the stability threshold, we additionally performed explicit DFT‐based convex hull evaluations for these candidates, confirming their thermodynamic stability against decomposition into competing phases (Table S4).

Finally, we identified two promising ferroelectric candidates, Ca_3_P_2_ and LiCdP (see Table 1). As shown in Figure 2c, their bandgaps fall within, or are close to, the targeted experimental bandgap window (1.4‐2.3 eV) [47]. Interestingly, the computed spontaneous polarization (*P*) value of LiCdP is higher than that of Al_1‐x_Sc_x_N [37, 38], which shows the highest *p* value reported so far. In contrast, the *p* value of Ca_3_P_2_ is much smaller than that of LiCdP but remains comparable to those of known ferroelectric materials. Furthermore, both compounds exhibit favorable Δ*E* values comparable to those of well‐known ferroelectrics, indicating efficient switching and strong device applications.

**TABLE 1:** DFT‐calculated ferroelectric properties of Ca_3_P_2_ and LiCdP. DFT‐calculated bandgaps (*E_gap_*), spontaneous polarizations (*P*), energy differences between polar and nonpolar structures (Δ*E*), and crystal‐likeness (CL) score values of Ca_3_P_2_ and LiCdP. The *E_gap_* and Δ*E* values in parenthesis correspond to the HSE06 *E_gap_* and r^2^SCAN Δ*E* values, respectively.

	Ca_3_P_2_	LiCdP
*E_gap_* (eV)	0.87 (1.58)	0.42 (1.13)
*P* (µC cm^−2^)	8.9	144.1
Δ*E* (meV f.u.^−1^)	99 (318)	138 (428)
CL score	0.813	0.503

**FIGURE 3:**
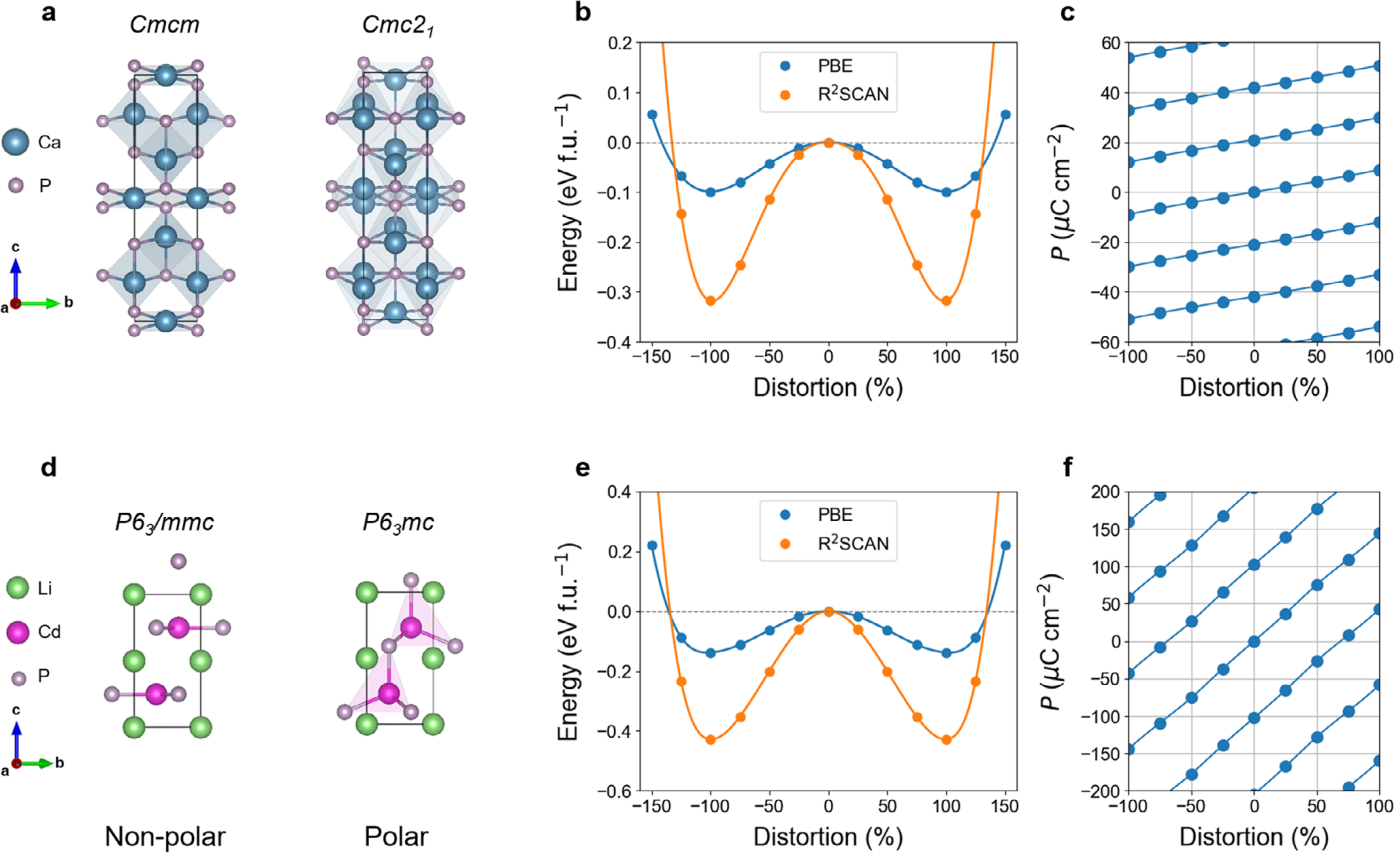
Structural and ferroelectric characterization of Ca_3_P_2_ and LiCdP. Nonpolar and polar structures of a) Ca_3_P_2_ and d) LiCdP, respectively. Double‐well potential profiles of b) Ca_3_P_2_ and e) LiCdP. The polarization (P) value as a function of the distortion from the –P polar structure through the centrosymmetric nonpolar structure to the +P polar structure for c) Ca_3_P_2_ and f) LiCdP. The calculated *p* values for a given distortion differ by multiples of the polarization quantum (***e*** R/***Ω***): 21.0 µC cm^−2^ for Ca_3_P_2_ and 102.5 µC cm^−2^ for LiCdP, where ***e*** is the charge of the electron, R is a lattice vector in the direction of polarization, and ***Ω*** is the volume of the unit cell.

Figure 3 shows the crystal structures and ferroelectric properties of *Cmc2_1_* Ca_3_P_2_ (Figure 3a–c) and *P6_3_mc* LiCdP (Figure 3d–f). The corresponding geometry information is provided in Figure S2. As shown in Figure 3a,d, atomic displacements along the *c*‐axis are responsible for the spontaneous *P* in both structures. The space groups of corresponding nonpolar structures are *Cmcm* for Ca_3_P_2_ and *P6_3_/mmc* for LiCdP, respectively. The double‐well potential profiles also show clear bistability (Figure 3b,e). The calculated Δ*E* values obtained from PBE are 99 meV f.u.^−1^ (f.u. indicates formula unit) for Ca_3_P_2_ and 138 meV f.u.^−1^ for LiCdP. These values are comparable to those of conventional ferroelectric materials, such as ∼55 meV f.u.^−1^ for BaTiO_3_ and ∼260 meV f.u.^−1^ for PbTiO_3_ [25]. This suggests that both candidates are likely to exhibit polarization switching under external electric fields. With r^2^SCAN functional [53], the double‐well potentials become deeper: 318 meV f.u.^−1^ for Ca_3_P_2_ and 428 meV f.u.^−1^ for LiCdP.

To better substantiate the photovoltaic and optoelectronic relevance of both Ca_3_P_2_ and LiCdP, we additionally calculated effective masses (m*), dielectric constants (ε), piezoelectric constants (γ), and carrier mobilities (μ) within the deformation potential (*D*) mechanisms. Computational details are shown in the Method section. These properties provide device‐relevant descriptors beyond bandgap and spontaneous polarization: the effective masses set the intrinsic limit for carrier mobility and diffusion length, the dielectric constants control Coulomb screening and exciton/charge separation, and the piezoelectric constants quantify the strength of electromechanical coupling and polarization–strain response that can influence built‐in fields and carrier collection. In addition, the carrier mobility estimated from the deformation‐potential framework provides a practical, comparative metric for charge‐transport capability under realistic scattering conditions. Taken together, these parameters enable a more quantitative device‐level assessment that is not possible from bandgap and polarization alone.

As shown in Table 2, both Ca_3_P_2_ and LiCdP show pronounced anisotropic in their dielectric, piezoelectric, and transport properties. Ca_3_P_2_ shows a nearly isotropic dielectric response, whereas LiCdP has the larger and more anisotropic permittivity, implying stronger electrostatic screening that can facilitate charge separation. LiCdP also generally exhibits larger γ values than Ca_3_P_2_, indicating stronger electromechanical coupling.

**TABLE 2:** Calculated dielectric (ε) tensors, piezoelectric (γ in e·Å) tensors, effective masses (m* in *m_e_* where *m_e_* is the mass of an electron), elastic constants (*C* in GPa), deformation potentials (*D* in eV), and mobilities (μ in cm^2^/(V s). For ε and γ, we only present non‐zero values. For LiCdP, we skipped $m_{22}^{*}$, *C*_22_, *D*_22_, and μ_22_ since it has the hexagonal crystal symmetry.

Property	Component	Ca_3_P_2_	LiCdP
ε	ε_11_	7.24	10.95
	ε_22_	7.26	10.95
	ε_33_	7.25	13.58
γ	$\gamma_{31}^{x}$	1.02	3.97
	$\gamma_{23}^{y}$	1.71	3.97
	$\gamma_{11}^{z}$	−0.14	2.31
	$\gamma_{22}^{z}$	−0.13	2.31
	$\gamma_{33}^{z}$	0.34	−2.58
m*	$m_{11}^{*}$ (*e*/*h*)	1.01 / 2.65	1.33 / 0.68
	$m_{22}^{*}$ (*e*/*h*)	0.32 / 2.27	—
	$m_{33}^{*}$ (*e*/*h*)	1.17 / 0.66	0.29 / 0.69
*C*	*C*_11_	24.83	19.301
	*C*_22_	24.45	—
	*C*_33_	99.86	112.31
*D*	*D*_11_	2.39/4.24	2.88/0.82
	*D*_22_	2.82/4.72	—
	*D*_33_	2.73/0.38	7.48/5.08
μ	μ_11_	257.19/7.36	69.22/4503.23
	μ_22_	3149.08/8.62	—
	μ_33_	552.54/117491.59	2790.19/679.90

Furthermore, the calculated m*, *D*, and μ values reveal clear direction‐ and carrier‐dependent transport advantages. Ca_3_P_2_ has a relatively light in‐plane m* for electrons (*e*) and correspondingly high in‐plane μ for *e*, while its hole (*h*) transport is particularly favorable along the out‐of‐plane direction. In contrast, LiCdP shows excellent in‐plane μ values for holes due to the comparatively small m* and modest *D* values for *h* along the in‐plane direction. It also supports the high μ value enabled by the small m* value along the out‐of‐plane direction. Overall, these results suggest that both materials can offer efficient carrier extraction and long diffusion lengths, but with distinct preferred transport directions that should be considered when proposing realistic photovoltaic/optoelectronic device architectures.

Additionally, we explicitly calculated the bulk photovoltaic (shift current) response (σ) of Ca_3_P_2_ and LiCdP using the Wannier90 package [54, 55] to directly assess whether the identified ferroelectric candidates can exhibit an intrinsic photovoltaic response. The shift current formalism describes the nonlinear photocurrent generated in non‐centrosymmetric crystals under uniform illumination and is widely used to evaluate bulk photovoltaic effects in ferroelectric and polar semiconductors [56, 57]. The calculated frequency‐dependent shift current spectra show finite bulk photovoltaic responses for Ca_3_P_2_, with the dominant tensor components, σ_*zzz*_, σ_*zxx*_, and σ_*zyy*_, confirming that its polar crystal symmetry supports an intrinsic bulk photovoltaic effect (Figure S3). Similarly, LiCdP exhibits nonzero tensor components, σ_*zzz*_, σ_*zyy*_, and σ_*yyz*_, consistent with its polar crystal symmetry (Figure S3). The pronounced anisotropy of the shift current response reflects the anisotropic electronic structure and polarization direction.

Interestingly, the crystal structure of LiCdP shown in Figure 3d corresponds to a wurtzite‐type CdP framework with Li atoms incorporated and was previously reported by Bennett et al. [58], although with different elemental compositions. Notably, LiCdP exhibits the lower Δ*E* value than other wurtzite‐type ferroelectric materials (LiMP, M = Be, Mg, Zn), while its spontaneous *p* value obtained from Berry‐phase calculations is the largest (144.1 µC cm^−2^). This value is even slightly higher than the experimental *p* value of the wurtzite Al_1‐_ *_x_* Sc*_x_* N [59], one of the highest‐polarization ferroelectrics reported to date. Thus, the identification of LiCdP represents a meaningful expansion of the wurtzite‐derived ferroelectric family. For Ca_3_P_2_, the calculated spontaneous *p* value is 8.9 µC cm^−2^ (Figure 3c,f) which is comparable to those of hexagonal rare‐earth ferrites [60, 61]. We also find that the crystal structure of Ca_3_P_2_ does not match any existing prototype as determined using XtalFinder [62]. This indicates that our generative framework can computationally identify and propose previously unexplored structural prototypes with strong ferroelectric potential, thereby expanding the accessible design space for ferroelectric materials.

To elucidate the origin of the unusually high polarization in LiCdP, we used DFT to analyze its atomic displacements and partial density of states (PDOS), benchmarking the results against Sc‐doped AlN. Specifically, we first fully optimized the crystal structure of ferroelectric Al_0.75_Sc_0.25_N (25% Sc‐doped AlN), as reported previously [59, 63], and present the optimized structure in Figure S4.

**FIGURE 4:**
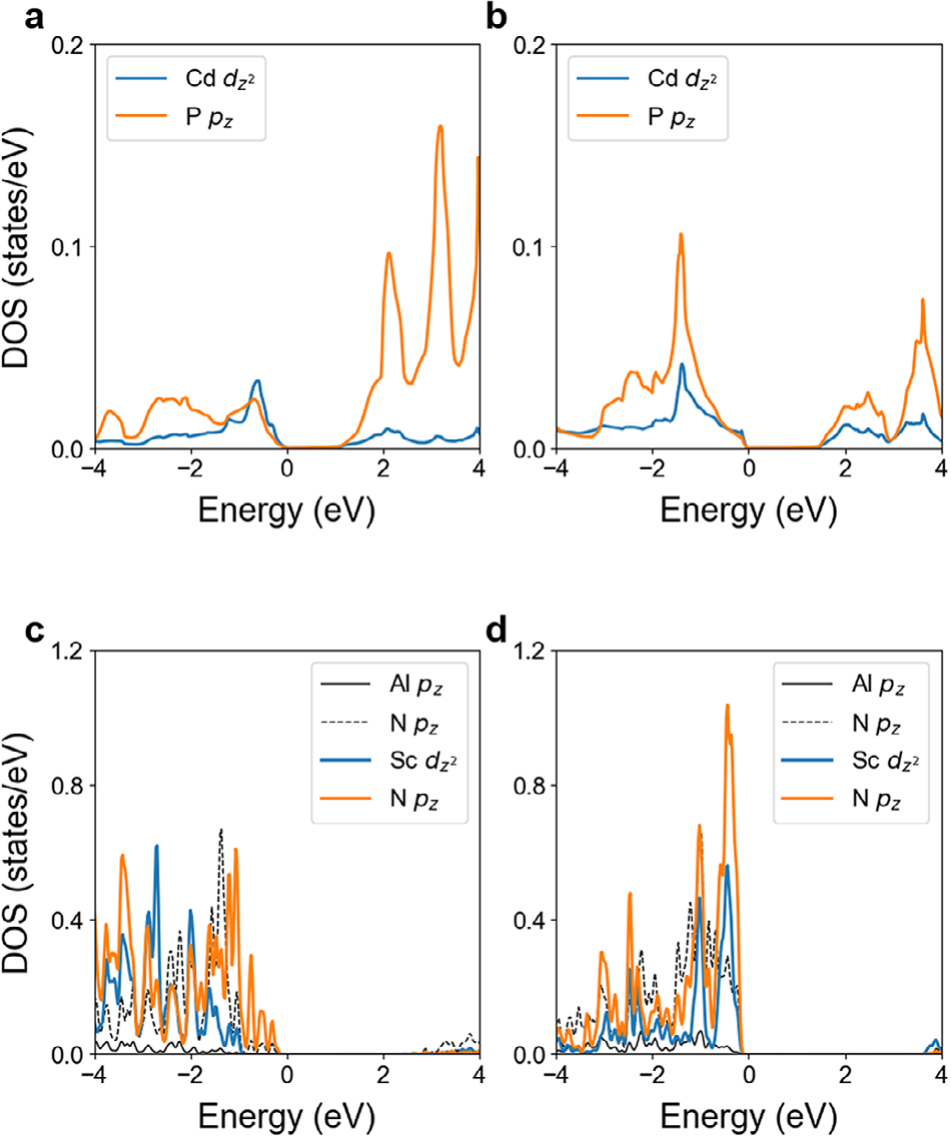
Calculated partial density of states (PDOS) of (a) nonpolar and (b) polar LiCdP, and of (c) nonpolar and (d) polar Al_0.75_Sc_0.25_N.

Using these optimized structures, we investigated the origin of ferroelectricity in LiCdP by comparing it with Al_0.75_Sc_0.25_N. Figure 4 presents the calculated PDOS of LiCdP alongside that of Al_0.75_Sc_0.25_N. In both cases, we can clearly see the $d_{z^{2}}-p_{z}$ orbital hybridization in the polar phase, consistent with the conventional electronic origin of ferroelectricity discussed for prototypical perovskites such as BaTiO_3_ and PbTiO_3_ [64, 65, 66]. Notably, in Al_0.75_Sc_0.25_N, the hybridization primarily involves Sc $3d_{z^{2}}$ and N 2*p_z_* states, whereas the Al 2*p_z_* and N 2*p_z_* states show much weaker mixing. This indicates that Sc substitution plays a key role in enabling the ferroelectric distortion in Al_0.75_Sc_0.25_N.

**FIGURE 5:**
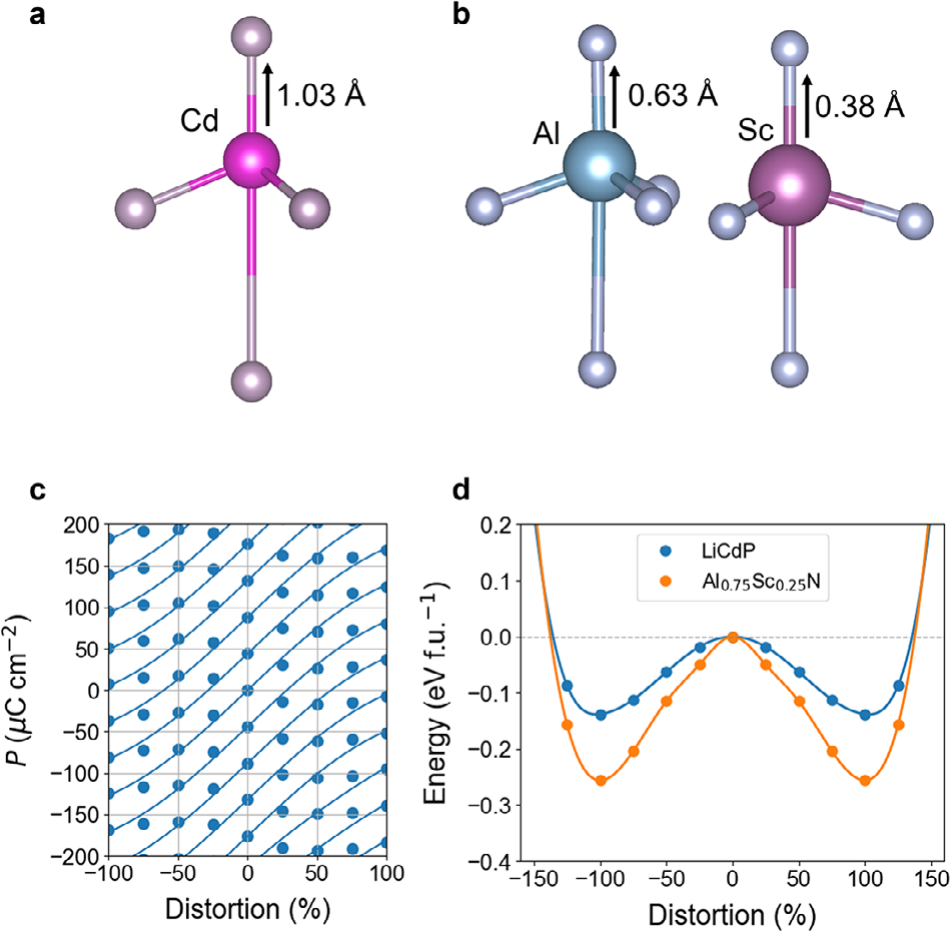
Off‐centering displacements in the polar structures: (a) Cd in LiCdP and (b) Al and Sc in Al_0.75_Sc_0.25_N. (c) *p* values for a given distortion differ by multiples of the polarization quantum ***e*** R/***Ω*** is 44.1 µC cm^−2^ for Al_0.75_Sc_0.25_N. (d) Double‐well potential energy profiles along the ferroelectric distortion coordinate for LiCdP and Al_0.75_Sc_0.25_N.

We then investigated the atomic displacements and calculated spontaneous polarization values. As shown in Figure 5a,b, the Cd displacement in LiCdP (1.03 Å) is larger than the Al (0.63 Å) and Sc (0.38 Å) displacements in Al_0.75_Sc_0.25_N. This larger displacement likely contributes to the slightly higher spontaneous polarization of LiCdP (144.1 µC cm^−2^) compared with Al_0.75_Sc_0.25_N (124.8 µC cm^−2^) (Figure 5c).

We also compared the double‐well barriers of the two materials. As shown in Figure 5d, LiCdP exhibits a slightly lower double‐well barrier than Al_0.75_Sc_0.25_N. It suggests that the coercive field required for ferroelectric polarization switching in LiCdP might be smaller than that in Al_0.75_Sc_0.25_N, which is consistent with the polarization–electric field (P–E) hysteresis loops calculated using the double‐well potentials and the Landau–Ginzburg–Devonshire (LGD) formalism in Figure S5 and Table S5 [67, 68].

As shown in Figure S5, we estimated intrinsic coercive fields by minimizing the LGD free energy functional under a uniform electric field, following established theoretical frameworks. This yields an intrinsic coercive field of 4.16 MV/cm for LiCdP, which is about eight times smaller than that of Al_0.75_Sc_0.25_N (32.5 MV/cm).

We emphasize that these intrinsic coercive fields are not intended to directly predict experimental switching thresholds because they neglect polarization switching via nucleation and domain‐wall motion—processes that typically lower the effective switching field in real materials. Nevertheless, the intrinsic values remain a useful comparative metric when evaluated consistently across materials.

Notably, for Al_0.75_Sc_0.25_N, which is known to possess a large intrinsic barrier, experimentally reported coercive fields are ∼4–7 MV/cm [69, 70, 71], nearly an order of magnitude lower than the intrinsic estimate (32.5 MV/cm). By applying a similar reduction factor to LiCdP, we estimate a practical coercive field on the order of ∼0.5–0.9 MV/cm [72, 73, 74, 75, 76, 77, 78]. This range is comparable to that of many conventional ferroelectrics and indicates that electric‐field‐induced switching in LiCdP could be is experimentally achievable.

To further assess the practical synthesizability of the shortlisted candidates, we evaluated synthesizability using positive‐unlabeled CGCNN (PU‐CGCNN) model [79]. In this model, the synthesizability is represented as crystal‐likeness (CL) scores. Ca_3_P_2_ yielded a CL score of 0.813, suggesting strong synthetic accessibility. LiCdP, however, exhibited a borderline CL score of 0.503, slightly above the empirical threshold of 0.5, indicating potential experimental synthesizability with substantial uncertainty (Table 1). Given the known limitations and possible false negatives of PU‐learning–based classifiers near the threshold [79], this result should be interpreted cautiously and regarded as motivation for further experimental validation rather than a conclusive prediction.

In addition, we explored possible synthesis routes for LiCdP by analyzing plausible chemical synthesis pathways for LiCdP using the ARROWS^3^ framework, which evaluates precursor‐dependent reaction energetics [80]. Among the examined pathways, the direct elemental synthesis (Li + Cd + P → LiCdP) was found to be energetically favorable, with reaction energies of approximately −60 meV/atom (Table S6). Experimental evidence for the feasibility of such elemental synthesis routes already exists. LiCdP (zinc‐blende structure, a different phase from our study) has been experimentally synthesized using elemental Li, Cd, and P precursors, as reported in ref. [81], in the context of Li(Cd,Mn)P diluted magnetic semiconductors. This shows that LiCdP in the phase identified in our study can form under appropriate chemical conditions despite its metastable nature. Taken together, these results suggest that LiCdP should be regarded as a metastable phase that may be experimentally accessible within a limited stability window under appropriate synthesis conditions.

**FIGURE 6:**
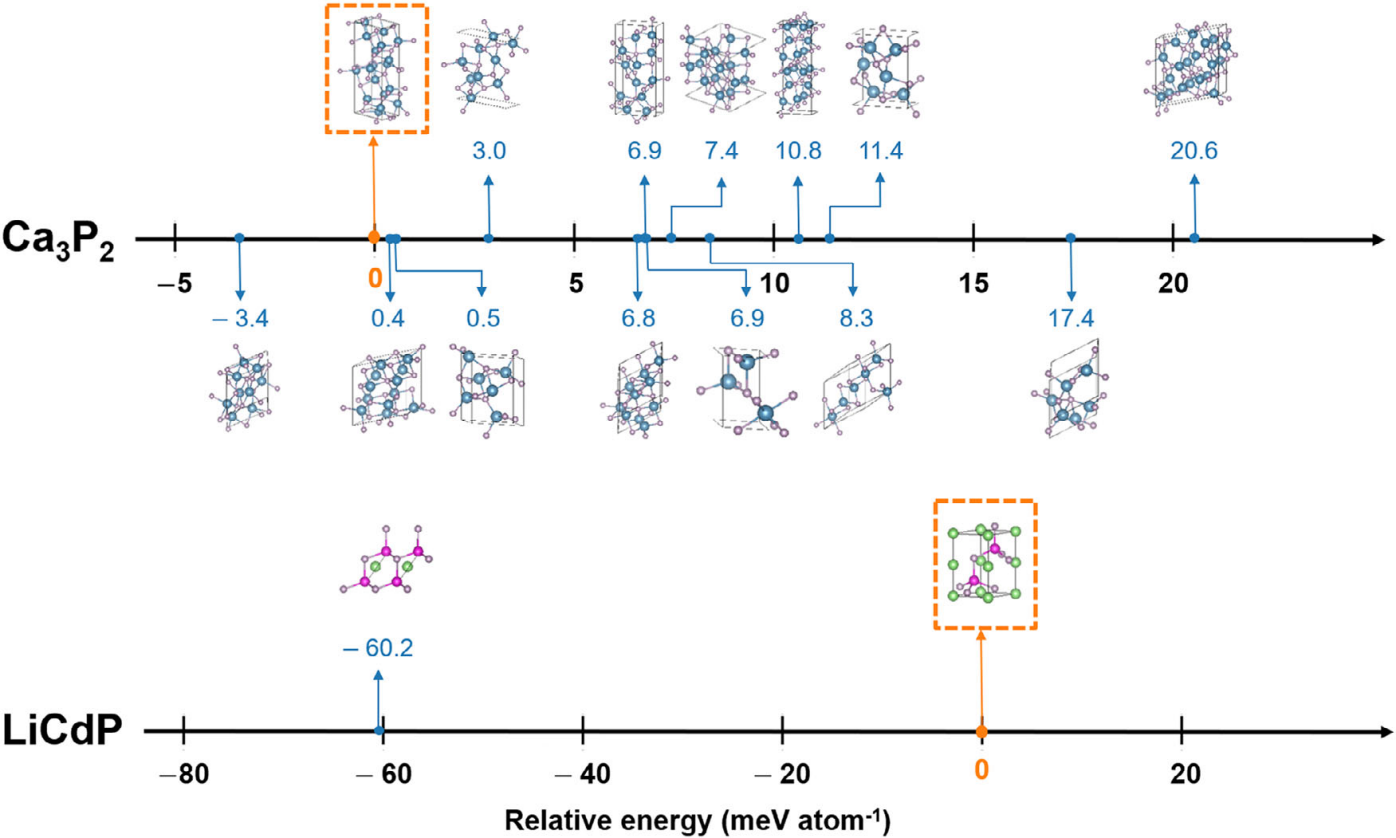
Crystal structure prediction results for Ca_3_P_2_ (up) and LiCdP (down). Multiple structures of the same composition were generated with the energy above hull plotted along the horizontal axis (lower values indicate greater stability). Each structure is annotated with its energy above hull. The discovered structures are highlighted by orange dashed lines.

As MatterGen explores a wide range of compositions, it may not sample each composition exhaustively and thus could miss the true ground‐state structures for the generated compositions. For the validation of the stability of both candidates, we performed the crystal structure prediction using the DiffCSP model [19]. A total of 4048 structures (1024 for each formula unit of 1–4) were generated, and the relative stabilities of ground state and metastable phases were quantified (Figure 6). Ca_3_P_2_ exhibits 13 distinct structural variants under 20 meV atom^−1^ with the identified candidate lying only 3 meV atom^−1^ above the most stable configuration. This small energy difference suggests a high likelihood of experimental synthesis of the discovered candidate. In contrast, the wurtzite LiCdP structure is 60 meV atom^−1^ above the ground state (zinc‐blende structure), which is relatively high. Nevertheless, as no other metastable structures are found within this energy range and given that several techniques have been reported to enable phase transitions between zinc‐blende and wurtzite structures [82, 83], the phase may still be synthesizable under appropriate conditions. We note that our calculated hull energies for Ca_3_P_2_ and LiCdP are ‐0.235 and ‐0.043 eV atom^−1^, respectively, which are much lower than those of other structures with the same compositions reported in the Materials Project (0.15 and 0.22 eV atom^−1^).

It is also worthy to noting that the low‐temperature crystal structure of Ca_3_P_2_ remains unknown even though this compound has been used in various applications, including as a rodenticide [84], and a precursor for phosphide compounds [85]. The available crystallographic information corresponds only to the high‐temperature phase: the crystal structure was reported under synthesis conditions at 1200°C [86], and X‐ray diffraction (XRD) data are available at 800–1000°C [87]. In contrast, both the lowest‐energy structure obtained from our calculations and the MatterGen‐discovered structure (a ferroelectric candidate) differ from these previously reported structures. Specifically, we find that the energy of our candidate structure is 46 meV per atom lower than that of the structure in ref. [86], and its XRD pattern also differs from that reported in ref. [87]. Notably, the structure in ref. [86] contains partially occupied sites; we accounted for this by constructing a supercell with two formula units. However, given the large energy difference, we consider it unlikely that the relative stability would be reversed. Therefore, our findings may represent the low‐temperature phase of Ca_3_P_2_, which requires further experimental verification.

We used uniform manifold approximation and projection (UMAP) [88] to confirm that the structures generated by MatterGen share the same distribution as the Alex‐MP20 dataset [42, 89] (Figure S6). The two identified candidates also fall within this range, indicating that the generative model successfully discovered new materials within the learned chemical space. (See the related text in the Supporting Information.)

### 2.2 Dynamic Stability and Band Structures

**FIGURE 7:**
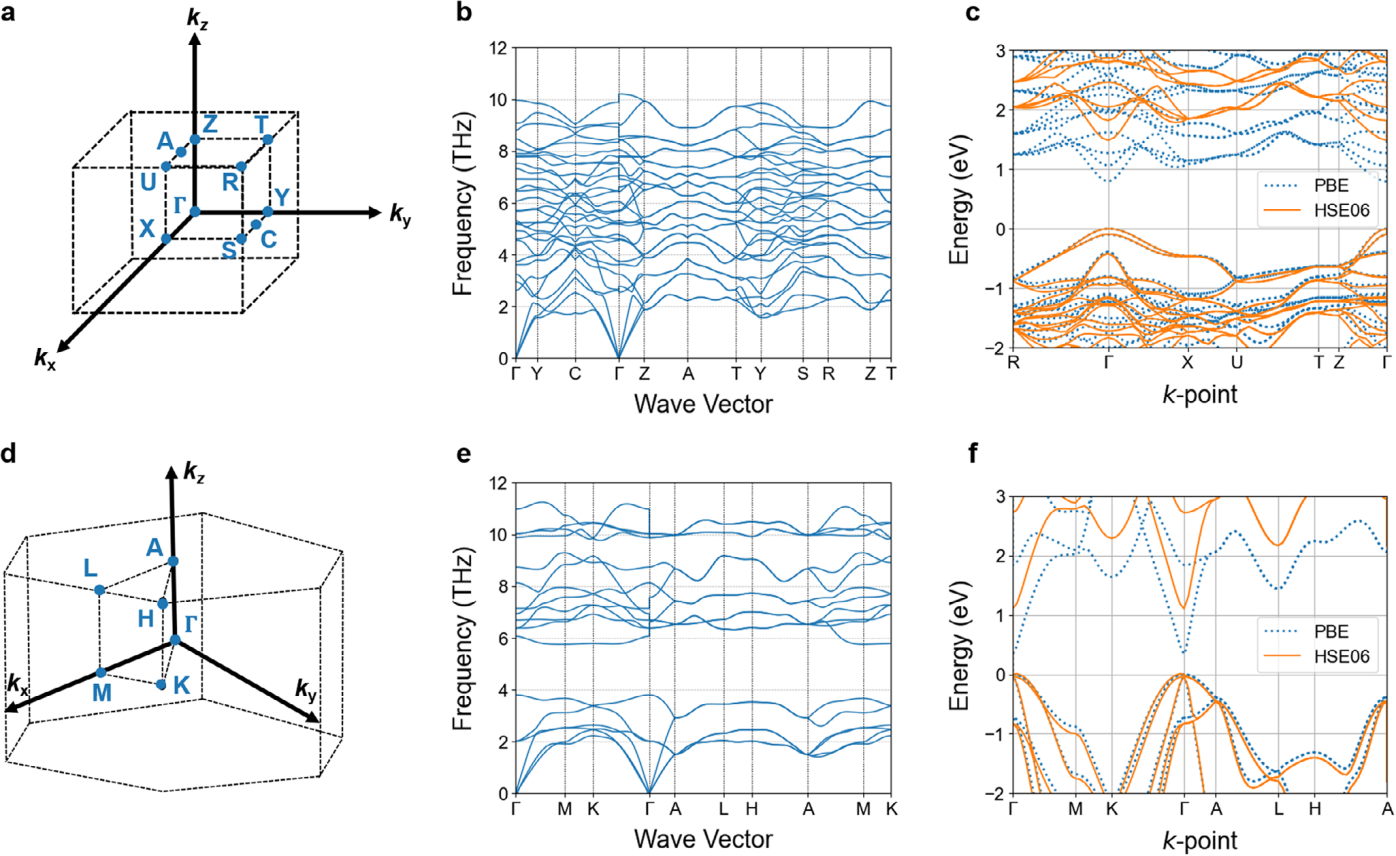
Brillouin zone and band structure analysis of Ca_3_P_2_ and LiCdP. 3‐dimensional schematic of the Brillouin zone of a) Ca_3_P_2_ and d) LiCdP. Phonon band dispersions of b) Ca_3_P_2_ and e) LiCdP. Electronic band structures of c) Ca_3_P_2_ and f) LiCdP.

Figure 7 shows the phonon and electronic band structures of Ca_3_P_2_ (Figure 7a–c) and LiCdP (Figure 7d–f). The phonon bands in Figures 7b,e exhibit no imaginary modes, indicating that the crystal structures of Ca_3_P_2_ and LiCdP are dynamically stable. We can also clearly see longitudinal optic mode–transverse optic mode (LO‐TO) splitting in both Ca_3_P_2_ and LiCdP, which is originated from dipoles in the crystal structures [90].

To further investigate the thermal stability of the candidate materials, we first performed ab initio molecular dynamics (AIMD) simulations to examine the thermal stability of the candidate materials. Accordingly, we conducted AIMD simulations under constant‐pressure and constant‐temperature (NPT) conditions at 700 K for 100 ps. The results are presented in Figures S7 and S8 for Ca_3_P_2_ and LiCdP, respectively. We find that both materials exhibit well‐converged energies and volumes throughout the simulations (Figures S7a,b and S8a,b). In addition, the final structures do not show any significant distortion compared to the initial structures (Figures S7c,d and S8c,d).

We also examined the relative stability between non‐polar and polar phases of these materials by calculating their Helmholtz free energies under varying temperatures (Figure S9). We find that, for both materials, the polar phase remains thermodynamically more stable than the corresponding non‐polar phase up to 1000 K. These results indicate that both materials remain structurally stable at elevated temperature.

Additionally, we calculated the band structures of Ca_3_P_2_ and LiCdP with the HSE06 functional because HSE06 generally provides an accurate prediction of inorganic bandgaps with a mean relative error of ‐2.61% for semiconductors [91]. As shown in Figures 5c,f, the calculated bandgaps of Ca_3_P_2_ and LiCdP are 1.58 and 1.13 eV, respectively (Table 1). These values fall within or near the targeted experimental bandgap window (1.4–2.3 eV) relevant to ferroelectric photovoltaic applications [47]. In addition, Ca_3_P_2_ shows a direct bandgap, whereas LiCdP shows a nearly direct bandgap. These results further indicate that Ca_3_P_2_ and LiCdP are promising materials for optoelectronic applications. A more detailed analysis of the optical properties of these two structures will be highly desirable in future work.

### 2.3 Current Scope and Future Perspectives

The MatterGen model used in this study was trained on structures from the Materials Project and Alexandria databases containing at most 20 atoms per unit cell [42, 89]. As a result, its applicability remains limited when exploring chemical spaces beyond this domain. In a previous study, we observed that diffusion‐based models perform well for material classes that are densely sampled in existing databases, such as oxides and nitrides [92]. In contrast, their performance degrades significantly for under‐represented material classes, including systems rich in rare‐earth elements or carbides, which are sparsely sampled in current training datasets. We also confirmed that structure generation becomes unreliable for larger unit cells exceeding 20 atoms. Therefore, a potential limitation of the present study is that MatterGen effectively explores only material classes on which it is trained. Addressing these limitations will require future development of diffusion models capable of handling under‐sampled chemical spaces and larger unit cells.

In addition, we performed extensive exploration by generating and screening a large number of structures (12,800 candidates) until promising materials were identified. While conditional generation could substantially reduce the number of required samples (ref. 16), such approaches rely on sufficiently large and reliable property‐labeled datasets, which are currently unavailable for the target property considered in this study (ferroelectricity). At this stage, unconditional generation therefore provides a practical means of exploring the design space. Importantly, the randomly generated structures accumulated in this work can serve as valuable training data for future conditional generation models, potentially enabling more efficient and targeted materials discovery in subsequent studies.

## 3 Conclusion

We have successfully designed novel ferroelectric structures, showing the potential of generative AI and high‐throughput screening techniques in accelerating the discovery of functional materials. By employing the MatterGen diffusion model, we generated a diverse pool of 12,800 inorganic crystal structures, which were systematically filtered through a multi‐fidelity screening pipeline. This pipeline integrated symmetry analysis, multiple ML models (MLIP, CGCNN, and PU‐CGCNN), and first‐principles calculations. Through this rigorous workflow, we found two previously unreported compounds (Ca_3_P_2_ and LiCdP) which exhibit exceptional ferroelectric properties. These including clear bistability in double‐well potential profiles with the small Δ*E* and sizable spontaneous *P* values. Moreover, both Ca_3_P_2_ and LiCdP are dynamically stable and possess the direct or nearly direct band structures with the relevant bandgap values well‐suited for ferroelectric photovoltaic applications. The crystal structure prediction with the composition‐fixed diffusion model further confirmed the thermodynamic competitiveness of the Ca_3_P_2_ and LiCdP structures with energies close to the convex hull, showing their experimental feasibility. Taken together, our generative AI‐driven workflow offers a scalable and efficient strategy for exploring vast chemical spaces, overcoming the limitations of traditional trial‐and‐error and high‐throughput screening methods. The discovery of Ca_3_P_2_ and LiCdP not only expands the list of photovoltaic ferroelectric materials but also sets a precedent for data‐driven materials design in other functional domains. This approach paves the way for the rapid identification of cutting‐edge ferroelectric materials.

## 4 Methods

### 4.1 Generative Models

For MatterGen [16] we used the mattergen_base configuration (unconditional base model pretrained on the Alex‐MP‐20 dataset [42, 89]). The dataset of all generated materials has been deposited on Zenodo [93]. For DiffCSP [19], we used the pretrained model trained on the MPTS‐52 database [94]. 1024 structures were generated for each formula unit from 1 to 4 (total 4048 structures). We used the SevenNet‐0 [44] pretrained model for the optimization and energy evaluation of generated crystals. The structures are relaxed using ASE module [95] until the maximum force becomes smaller than 0.05 eV/Å.

### 4.2 DFT Calculations

To investigate the ferroelectric properties, we performed density functional theory (DFT) calculations using the projector augmented‐wave (PAW) method [96, 97] as implemented in the Vienna ab initio Simulation Package (VASP) [98, 99, 100]. To address the well‐known bandgap underestimation and soft phonon features of the PBE functional [48], we employed the HSE06 hybrid functional [101] for band structure calculations and the r^2^SCAN functional [53] for double‐well potential calculations. For structural optimization, we used the PBE functional with a plane‐wave cutoff energy of 520 eV and Monkhorst–Pack k‐point meshes of 7 × 2 × 4 for Ca_3_P_2_ and 7 × 7 × 4 for LiCdP. Ionic positions were relaxed until the Hellmann–Feynman forces were smaller than 0.001 eV Å^−1^. The following valence electron configurations were explicitly treated: Cd (4d^10^5s^2^), Ca (3s^2^3p^6^4s^2^), P (3s^2^3p^3^), and Li (2s^1^). For Berry‐phase polarization calculations, the electronic self‐consistency threshold was set to 10^−^^7^ eV. All crystal structure images shown in the figures were generated using the VESTA program [102].

The elastic moduli were obtained from the generalized form of Hooke's law which relates stress σ to strain ε:

$$\sigma_{i}=C_{\mathrm{ij}}\varepsilon_{j}$$

where C_ij_ is the single crystal elastic tensor in the Voigt notation. We computed *C_ij_* as follows: i) fully relax the unit‐cell lattice parameters and atomic positions; ii) apply strains to the optimized cell along the *x*‐, *y*‐, and *z*‐axes for Ca_2_P_3_ and along the *x*‐ and *z*‐axes for LiCdP. For each strain mode, seven deformations were considered: 0%, ±0.5%, ±1% and ±1.5%; iii) for each deformed lattice, relax the internal atomic coordinates while keeping the strained lattice parameters fixed; and (iv) obtain *C_ij_* by linear least‐squares fitting of the stress–strain data based on the above relation.

The charge carrier mobility µ_*ij*_ was calculated using the deformation‐potential theory, in which carrier‐phonon coupling in the acoustic regime is assumed to be the dominant scattering mechanism:

$$\;\mu_{ij}=\frac{{\left(8\pi \right)}^{1/2}\hbar^{4}eC_{ij}}{3{\left(m_{ij}^{*}\right)}^{5/2}{\left(k_{B}T\right)}^{3/2}{D_{ij}}^{2}}$$

where *e*, ℏ, *k_B_*, and *T* are the charge, the Plank constant divided by 2π, and the Boltzmann constant, and the temperature, respectively. We set *T*  =  300*K*. $m_{ij}^{*}$ was obtained from the numerical second derivative of the band dispersion, ∂^2^ *E*(*k*)/∂*k* ^2^, using eleven eigenvalues near the valence (conduction) band maxima (minima) around the 𝛤‐point [0, 0, 0]. Specifically, we sampled the band energies along 𝛤 → *X* = [0.5, 0, 0], 𝛤 → *Y* = [0, 0.5, 0], and 𝛤 → *Z* = [0, 0, 0.5] for Ca_3_P_2_ and along 𝛤 → *X* = [0.5, 0, 0] and 𝛤 → *Z* = [0, 0, 0.5] for LiCdP using 20 k‐points between the endpoints in each direction. *D_ij_* was extracted from the linear dependence of the relevant band‐edge energy on uniaxial strain. For each strain mode, seven deformations were considered: 0%, ±0.5%, ±1% and ±1.5% along the *x*‐, *y*‐, and *z*‐axes for Ca2P3 and along the *x*‐ and *z*‐axes for LiCdP. All eigenvalues were aligned with the P 1*s* core level.

### 4.3 Shift Current Calculations

The shift current responses of Ca_3_P_2_ and LiCdP were calculated using the Wannier90 [54, 55], interfaced with Quantum ESPRESSO [103], within the PBE exchange–correlation framework [48] and ultrasoft pseudopotentials. Plane‐wave cutoff energies of 60 Ry (480 Ry for charge density) were employed. Non‐self‐consistent calculations were performed with fixed occupations and symmetry disabled to ensure compatibility with Wannier interpolation. Maximally localized Wannier functions were constructed using the disentanglement procedure with the Wannier90 package [54, 55]. For Ca_3_P_2_, 96 bands were included, and 44 Wannier functions were used with an outer energy window of −10 to 20 eV. For LiCdP, 60 bands and 34 Wannier functions were used with an outer window of −10 to 24 eV. The shift current tensor was evaluated using the Berry‐phase formalism on dense interpolation *k*‐meshes, with convergence verified up to 10×3×8 for Ca_3_P_2_ and 24 × 24 × 16 for LiCdP, over a photon energy range of 0–12 eV.

### 4.4 AIMD Simulations

NPT AIMD simulations were performed using a Langevin thermostat. The friction coefficients for the atomic and lattice degrees of freedom were set to 1.0 ps^−1^, and the fictitious cell mass was set to 1000 amu. AIMD simulations were carried out using 3 × 2 × 1 (120 atoms) and 3 × 3 × 2 (108 atoms) supercells for Ca_3_P_2_ and LiCdP, respectively. Phonon calculations were performed using 2 × 2 × 1 (80 atoms) and 3 × 3 × 2 (108 atoms) supercells for Ca_3_P_2_ and LiCdP, respectively. Phonon spectra and Helmholtz free‐energy calculations were performed using the Phonopy code [104]. For the phonon and Helmholtz free‐energy calculations, we employed the local‐density approximation (LDA) exchange–correlation functional, which is known to provide accurate vibrational properties for many inorganic solids [105]. Using LDA, the energy differences *ΔE* were 0.201 and 0.404 eV per formula unit for Ca_3​_P_2_​ and LiCdP, respectively. These values are close to those obtained with r^2^SCAN (Table 1), supporting the validity of using LDA for the Helmholtz free‐energy analysis.

## Author Contributions

B.C.Y., S.K., and J.‐H.L. conceived the study. B.C.Y. developed the screening workflow and performed the analyses under the supervision of S.K. and J.‐H.L. H.‐J. Lee performed numerical calculations to obtain the coercive fields. B.C.Y. wrote the initial version of the manuscript. S.K. and J.‐H.L. revised the manuscript. All authors discussed the results and contributed to the final version of the manuscript.

## Conflicts of Interest

The authors declare no conflicts of interest.

## Supporting information




**Supporting File**: advs74931‐sup‐0001‐SuppMat.docx.

## Data Availability

The dataset of all generated materials has been deposited on Zenodo [93]. Structural data of the newly identified ferroelectric candidates are provided in the Supporting Information. The codes for executing the pipeline illustrated in Figure 1 is freely available at https://github.com/yobc401/FerroGen and can also be obtained from the corresponding authors upon reasonable request.
