# Supplementary material for: Diffusion–Model–Driven Discovery of Ferroelectrics for Photocurrent Applications
Source: Adv Sci (Weinh). 2026 Mar 28;13(31):e22108. doi: 10.1002/advs.202522108 (PMC13587980; doi:10.1002/advs.202522108)
Supplement: Supplementary file 1 [file ADVS-13-e22108-s001.docx]

**Supporting Information for**

**Diffusion–Model–Driven Discovery of Ferroelectrics for Photocurrent Applications**

Byung Chul Yeo^a^ , Hyun-Jae Lee^b^, Sungwoo Kang^b,c*^, and Jung-Hoon Lee^b,d*^

^a^Department of Energy Resources Engineering, Pukyong National University, Busan 48513, Republic of Korea

^b^Computational Science Research Center, Korea Institute of Science and Technology (KIST), Seoul 02792, Republic of Korea

^c^Division of Nanoscience and Technology, KIST School, University of Science and Technology (UST), Seoul 02792, Republic of Korea

^d^KU-KIST Graduate School of Converging Science and Technology, Korea University, Seoul 02841, Republic of Korea

* Corresponding author: [sung.w.kang@kist.re.kr](mailto:sung.w.kang@kist.re.kr), [jhlee84@kist.re.kr](mailto:jhlee84@kist.re.kr)

Table S1. 42 polar space groups listed by the space group number along with their international notation and corresponding crystal system.

| Space group number | Space group notation | Crystal system |
| --- | --- | --- |
| 4 | P2_1_ | Monoclinic |
| 5 | C2 | Monoclinic |
| 7 | Pc | Monoclinic |
| 8 | Cm | Monoclinic |
| 9 | Cc | Monoclinic |
| 26 | Pmc2_1_ | Orthorhombic |
| 29 | Pca2_1_ | Orthorhombic |
| 31 | Pmn2_1_ | Orthorhombic |
| 32 | Pba2 | Orthorhombic |
| 33 | Pna2_1_ | Orthorhombic |
| 35 | Cmm2 | Orthorhombic |
| 36 | Cmc2_1_ | Orthorhombic |
| 37 | Ccc2 | Orthorhombic |
| 38 | Aem2 | Orthorhombic |
| 39 | Abm2 | Orthorhombic |
| 40 | Amm2 | Orthorhombic |
| 41 | Aba2 | Orthorhombic |
| 43 | Fdd2 | Orthorhombic |
| 44 | Imm2 | Orthorhombic |
| 45 | Ama2 | Orthorhombic |
| 46 | Ima2 | Orthorhombic |
| 76 | P4 | Tetragonal |
| 79 | I4 | Tetragonal |
| 80 | I4_1_ | Tetragonal |
| 100 | P4bm | Tetragonal |
| 102 | P4_2_bc | Tetragonal |
| 103 | P4cc | Tetragonal |
| 105 | P4nc | Tetragonal |
| 107 | I4mm | Tetragonal |
| 108 | I4cm | Tetragonal |
| 110 | I4cm | Tetragonal |
| 143 | P3 | Trigonal |
| 146 | R3 | Trigonal |
| 156 | P3m1 | Trigonal |
| 157 | R3c | Trigonal |
| 158 | R3m | Trigonal |
| 159 | R3c | Trigonal |
| 160 | R3c | Trigonal |
| 161 | R3m | Trigonal |
| 173 | P6_3_ | Hexagonal |
| 185 | P6_3_cm | Hexagonal |
| 186 | P6_3_mc | Hexagonal |

Figure S1. Periodic table annotated with allowed oxidation states.^[1]^ Integers shown above each element symbol indicate the admissible states when testing charge neutrality.

Table S2. List of screened structures with the energy above hull (E.A.H.) less than 50 meV atom^-1^, CGCNN-predicted band gaps ($\boldsymbol{E}_{\boldsymbol{gap}}$), and crystal-likeness (CL) score.

| No. | Space group | Chemical formula | E.A.H.  (eV atom^-1^) | $E_{gap}$  (eV) | CL score |
| --- | --- | --- | --- | --- | --- |
| 1 | P3m1 | F3KSe | -0.277 | 2.073 | 0.654 |
| 2 | Cmc2_1_ | Ca3P2 | -0.235 | 1.505 | 0.813 |
| 3 | Aea2 | OS | -0.178 | 3.848 | 0.912 |
| 4 | Cc | GeH3Rb | -0.165 | 3.283 | 0.758 |
| 5 | R3m | CClTmY | -0.130 | 0.436 | 0.358 |
| 6 | C2 | F3LiPd | -0.103 | 2.387 | 0.927 |
| 7 | R3m | CHoITm | -0.103 | 0.799 | 0.438 |
| 8 | Pmc2_1_ | MoN3Tb | -0.102 | 1.721 | 0.038 |
| 9 | C2 | AuCl3Pd | -0.096 | 1.136 | 0.884 |
| 10 | R3m | BiRbTe2 | -0.090 | 0.966 | 0.666 |
| 11 | Cm | AgHSe | -0.084 | 1.009 | 0.772 |
| 12 | Amm2 | Cs2PtSb2 | -0.078 | 0.436 | 0.980 |
| 13 | I4mm | F2OTlV | -0.067 | 2.991 | 0.356 |
| 14 | Cm | BrErO8Se2 | -0.065 | 2.706 | 0.667 |
| 15 | Cm | Cl6CuInPd | -0.061 | 0.418 | 0.804 |
| 16 | P3m1 | I3SV | -0.049 | 0.495 | 0.892 |
| 17 | Amm2 | PtRb2Sb2 | -0.045 | 0.613 | 0.829 |
| 18 | P6_3_mc | CdLiP | -0.043 | 0.936 | 0.503 |
| 19 | Cmc2_1_ | Br4CoTl2 | -0.038 | 0.904 | 0.491 |
| 20 | R3m | BrHHfS | -0.036 | 2.094 | 0.799 |
| 21 | Cc | BiF7NaTl | -0.028 | 1.605 | 0.439 |
| 22 | Pmn2_1_ | Cu2S3Si | -0.027 | 1.172 | 0.802 |
| 23 | C2 | N7Nd3W3 | -0.021 | 0.960 | 0.014 |
| 24 | Imm2 | AsH4IrLa | -0.018 | 0.459 | 0.854 |
| 25 | Cmc2_1_ | Ce2N5TaTi | -0.015 | 0.696 | 0.188 |
| 26 | R3m | LiLuSe2 | -0.014 | 1.243 | 0.720 |
| 27 | C2 | AgF3Zn | -0.013 | 0.391 | 0.006 |
| 28 | Cm | Dy4K3LiSe8 | -0.012 | 1.809 | 0.435 |
| 29 | R3m | HfNa2O4Pb | -0.012 | 3.946 | 0.207 |
| 30 | R3m | AsLaSSm | -0.011 | 1.047 | 0.187 |
| 31 | R3m | FHMnO | -0.010 | 2.093 | 0.140 |
| 32 | P3m1 | Au2LiSb2Tb | -0.010 | 0.404 | 0.910 |
| 33 | R3m | PdSe | -0.008 | 0.371 | 0.924 |
| 34 | C2 | Br6Fe2Li2 | -0.008 | 0.541 | 0.536 |
| 35 | Cmc2_1_ | AgCdCl5K2 | -0.007 | 2.349 | 0.848 |
| 36 | Cm | F8Li2OW2 | -0.006 | 3.077 | 0.194 |
| 37 | R3m | CSeTiZr | -0.006 | 0.434 | 0.390 |
| 38 | C2 | CuSn2Te3 | -0.004 | 0.384 | 0.260 |
| 39 | Cmc2_1_ | Ce2CuSe5Tb | -0.002 | 0.688 | 0.766 |
| 40 | I4mm | HoOSbTm | -0.001 | 3.089 | 0.119 |
| 41 | Cmc2_1_ | AgBr5PbTl2 | -0.001 | 2.670 | 0.631 |
| 42 | P21 | HORb | 0.000 | 3.493 | 0.697 |
| 43 | P6_3_mc | NaSbZn | 0.000 | 0.473 | 0.786 |
| 44 | P6_3_mc | NiS | 0.000 | 1.103 | 0.504 |
| 45 | Cm | FIO4Rb2 | 0.001 | 1.927 | 0.423 |
| 46 | I4mm | OSbSmY | 0.004 | 2.413 | 0.057 |
| 47 | R3m | BrHS | 0.005 | 2.776 | 0.833 |
| 48 | Pc | AlGaLi2S4 | 0.005 | 2.883 | 0.629 |
| 49 | Pc | BeLi2O4Si | 0.006 | 5.448 | 0.809 |
| 50 | Cm | Cs2Cu2I4 | 0.006 | 2.048 | 0.530 |
| 51 | Imm2 | Cu2Na2STe | 0.006 | 0.393 | 0.966 |
| 52 | C2 | F4LiRbTl2 | 0.006 | 1.604 | 0.395 |
| 53 | Cmc2_1_ | AlHO2 | 0.006 | 5.175 | 0.591 |
| 54 | Cmc2_1_ | Ba2HfHgSe5 | 0.006 | 1.117 | 0.966 |
| 55 | Cm | AgHo3Sb2Se0 | 0.007 | 0.631 | 0.553 |
| 56 | Cm | F6HfHg2 | 0.009 | 3.080 | 0.485 |
| 57 | Amm2 | AsCdH2Rb3 | 0.010 | 1.493 | 0.953 |
| 58 | R3m | Co2Mn2Se4 | 0.011 | 0.469 | 0.148 |
| 59 | Aem2 | BrH | 0.011 | 4.282 | 0.631 |
| 60 | Cm | I2In2S2 | 0.011 | 1.653 | 0.830 |
| 61 | Cm | Ba4I9K | 0.012 | 3.319 | 0.422 |
| 62 | Pmn2_1_ | CdHgS2 | 0.014 | 1.603 | 0.216 |
| 63 | R3m | CEuO | 0.014 | 0.832 | 0.827 |
| 64 | C2 | C2Na2Nd2O6 | 0.014 | 4.602 | 0.597 |
| 65 | Cmc2_1_ | InSe5SmSr2 | 0.015 | 1.049 | 0.932 |
| 66 | Cm | Cl3PbTl | 0.015 | 2.810 | 0.232 |
| 67 | R3m | GeTe | 0.015 | 0.371 | 0.598 |
| 68 | Cmc2_1_ | GaSbSe5Sr2 | 0.015 | 1.303 | 0.935 |
| 69 | Cm | Ce4I3N3P2 | 0.015 | 0.567 | 0.889 |
| 70 | Amm2 | BiCl5InTl | 0.016 | 2.899 | 0.886 |
| 71 | P3m1 | DyLiSe2 | 0.016 | 1.824 | 0.216 |
| 72 | Cm | I6MnRb2Tl | 0.017 | 2.023 | 0.808 |
| 73 | C2 | KNaO3Te | 0.018 | 3.650 | 0.963 |
| 74 | Cm | Br3In3 | 0.018 | 1.504 | 0.824 |
| 75 | R3m | BiIn2NaS4 | 0.019 | 1.281 | 0.292 |
| 76 | P3m1 | BaSbTl | 0.020 | 0.508 | 0.528 |
| 77 | I4mm | AgBr3RbTl | 0.021 | 2.290 | 0.804 |
| 78 | Cmc2_1_ | AlHoPb2Se5 | 0.021 | 1.567 | 0.653 |
| 79 | R3m | HgNbSe2 | 0.022 | 0.420 | 0.557 |
| 80 | Cmc2_1_ | Cl3MnNa | 0.022 | 1.806 | 0.913 |
| 81 | Cm | Cl6Pb2Tl2 | 0.023 | 3.049 | 0.601 |
| 82 | Cc | CdFeKO3 | 0.024 | 2.066 | 0.184 |
| 83 | R3m | CsHoO2 | 0.024 | 3.155 | 0.318 |
| 84 | Pmn2_1_ | I3SrTl | 0.024 | 3.052 | 0.343 |
| 85 | Cm | Br9Cs4CuTl4 | 0.024 | 2.461 | 0.917 |
| 86 | R3m | PrRbS2 | 0.024 | 2.365 | 0.949 |
| 87 | Cc | AsF5 | 0.025 | 4.810 | 0.924 |
| 88 | P3m1 | Cl6Pb3 | 0.025 | 2.894 | 0.564 |
| 89 | Cm | IrNd2O8Y2 | 0.025 | 0.388 | 0.255 |
| 90 | Cmc2_1_ | F4MgSr | 0.026 | 6.590 | 0.834 |
| 91 | P3m1 | HgNbSe2 | 0.027 | 0.400 | 0.649 |
| 92 | R3m | CSV2 | 0.027 | 0.446 | 0.654 |
| 93 | Cmc2_1_ | ClILi2 | 0.028 | 4.289 | 0.497 |
| 94 | Cmc2_1_ | FeO5Sr2Tm | 0.028 | 2.217 | 0.536 |
| 95 | Cm | Cl4S2Sn4 | 0.029 | 2.034 | 0.535 |
| 96 | Cm | BiLiPb4Te6 | 0.029 | 0.409 | 0.064 |
| 97 | R3m | CdSe2Zn | 0.029 | 0.663 | 0.680 |
| 98 | Cmc2_1_ | S5SnTl2Zr | 0.029 | 1.885 | 0.905 |
| 99 | Cm | CuFHg3Rb3 | 0.030 | 0.551 | 0.651 |
| 100 | R3m | DyO2Rb | 0.030 | 3.805 | 0.755 |
| 101 | Cm | CsF7FeK3 | 0.031 | 4.313 | 0.926 |
| 102 | Cm | RbSe8Ta3 | 0.032 | 0.413 | 0.772 |
| 103 | Cm | Na3PbS4Tb | 0.032 | 2.348 | 0.257 |
| 104 | Cm | F6Na3Y | 0.032 | 6.694 | 0.548 |
| 105 | R3m | KLiNaSb | 0.032 | 1.900 | 0.943 |
| 106 | Cmc2_1_ | GaLiNd2Se5 | 0.033 | 1.259 | 0.256 |
| 107 | Cmc2_1_ | Ce2GaN5Zr | 0.033 | 1.585 | 0.574 |
| 108 | Cc | Br3LiRb2 | 0.033 | 4.319 | 0.486 |
| 109 | R3m | AsLuPY | 0.033 | 0.379 | 0.876 |
| 110 | Cm | K2Li2S4Zn2 | 0.033 | 2.421 | 0.320 |
| 111 | Pmn2_1_ | K2S3Zr | 0.033 | 2.937 | 0.334 |
| 112 | Cm | Br5In3K2 | 0.034 | 2.093 | 0.589 |
| 113 | Cm | I9InK4Li4 | 0.034 | 3.323 | 0.629 |
| 114 | Cm | AsClH2K2 | 0.035 | 3.870 | 0.881 |
| 115 | Cmc2_1_ | Ba2GePbSe5 | 0.035 | 1.388 | 0.951 |
| 116 | Ama2 | AsCsS3 | 0.035 | 1.447 | 0.966 |
| 117 | Imm2 | As3Cs3HgI5 | 0.035 | 1.420 | 0.216 |
| 118 | R3m | KSbZn | 0.035 | 0.556 | 0.919 |
| 119 | Cm | Li2Pb2Rb2Se4 | 0.036 | 2.145 | 0.932 |
| 120 | Cm | K2Se6Sm2Tb2 | 0.036 | 1.953 | 0.721 |
| 121 | Cmc2_1_ | Sm2SnTe4 | 0.036 | 0.609 | 0.407 |
| 122 | Cm | N5TaTi5 | 0.037 | 0.382 | 0.195 |
| 123 | P3m1 | ScTe2Tl | 0.037 | 0.373 | 0.834 |
| 124 | P3m1 | Cr2HgO4Rb2 | 0.037 | 1.831 | 0.776 |
| 125 | R3m | PbSSeSn | 0.037 | 1.084 | 0.234 |
| 126 | Cc | AgBeErF6 | 0.037 | 2.196 | 0.041 |
| 127 | P3m1 | CoSe2Tl | 0.038 | 0.433 | 0.637 |
| 128 | Cm | Cu2I7KSr2 | 0.038 | 2.063 | 0.692 |
| 129 | Cm | Na4Se6Sn2 | 0.039 | 0.652 | 0.937 |
| 130 | Cmc2_1_ | Ho2N5SiTa | 0.039 | 2.191 | 0.605 |
| 131 | Pna2_1_ | Br2CuK | 0.040 | 2.058 | 0.940 |
| 132 | Cm | Ag3I6Sc | 0.040 | 2.090 | 0.394 |
| 133 | Imm2 | Cl4CsPbRb | 0.041 | 3.518 | 0.281 |
| 134 | Amm2 | K4O2Se | 0.042 | 1.564 | 0.737 |
| 135 | Amm2 | Br6Sn2Tl2 | 0.042 | 1.348 | 0.401 |
| 136 | Cmc2_1_ | Br2LiTl | 0.043 | 3.011 | 0.785 |
| 137 | C2 | Ag2I3Tl | 0.043 | 2.715 | 0.261 |
| 138 | Cmc2_1_ | GePb3S5 | 0.043 | 1.319 | 0.875 |
| 139 | R3m | Br4InK | 0.044 | 2.748 | 0.462 |
| 140 | R3m | F5KNaSc | 0.045 | 5.764 | 0.533 |
| 141 | R3m | CsSSb | 0.046 | 2.313 | 0.999 |
| 142 | Cm | DySb6Sc2Y3 | 0.046 | 0.467 | 0.835 |
| 143 | Cm | Ba4Ho3O0Tl | 0.048 | 2.370 | 0.388 |
| 144 | Cm | Br6Cu2Sn2 | 0.049 | 1.481 | 0.652 |
| 145 | Cm | InRb2Sb3Tl4 | 0.049 | 0.725 | 0.801 |

Table S3. DFT-calculated PBE band gaps ($\boldsymbol{E}_{\boldsymbol{gap}}$) and energy differences ($\boldsymbol{\Delta E}$) between polar and nonpolar structures ($\boldsymbol{\Delta E}$ in eV f.u.^-1^) of 11 candidates.

| No. | Space group | Chemical formula | $E_{gap}$  (eV) | $\Delta E$  (eV f.u.^-1^) |
| --- | --- | --- | --- | --- |
| 1 | Cmc2_1_ | Ca_3_P_2_* | 0.874 | 0.099 |
| 2 | R3m | LiLuSe_2_ | - | Non-switchable |
| 3 | Pmn2_1_ | Cu_2_S_3_Si | Metallic | - |
| 4 | C2 | AuCl_3_Pd | Metallic | - |
| 5 | P6_3_mc | NiS | Metallic | - |
| 6 | Cm | AgHSe | 0.775 | 0.647 |
| 7 | R3m | BiRbTe_2_ | - | Non-switchable |
| 8 | P6_3_mc | LiCdP* | 0.423 | 0.138 |
| 9 | Cmc2_1_ | Ce_2_CuSe_5_Tb | Metallic | - |
| 10 | Amm2 | PtRb_2_Sb_2_ | Metallic | - |
| 11 | C2 | Br_6_Fe_2_Li_2_ | 1.557 | 0.689 |

Table S4. Comparison between DFT– and SevenNet-0–calculated total energies for 11 candidate compounds, including the absolute deviation in total energy ($\left| \boldsymbol{E}_{\boldsymbol{tot,DFT}}\boldsymbol{-}\boldsymbol{E}_{\boldsymbol{tot,SevenNet-0}} \right|$) and the corresponding DFT-calculated energy above convex hull (E.A.H.[DFT]).

| No. | Chemical formula | $\left\vert E_{tot,DFT}-E_{tot,SevenNet-0} \right\vert$  (eV/atom) | E.A.H.[DFT]  (eV atom^-1^) |
| --- | --- | --- | --- |
| 1 | Ca_3_P_2_* | 0.030 | -0.204 |
| 2 | LiLuSe_2_ | 0.005 | -0.009 |
| 3 | Cu_2_S_3_Si | 0.009 | -0.018 |
| 4 | AuCl_3_Pd | 0.060 | -0.033 |
| 5 | NiS | 0.043 | 0.054 |
| 6 | AgHSe | 0.017 | -0.056 |
| 7 | BiRbTe_2_ | 0.017 | -0.073 |
| 8 | LiCdP* | 0.004 | -0.046 |
| 9 | Ce_2_CuSe_5_Tb | 0.007 | -0.002 |
| 10 | PtRb_2_Sb_2_ | 0.041 | 0.006 |
| 11 | Br_6_Fe_2_Li_2_ | 0.005 | 0.019 |

**(a)**

Ca3P2_nonpolar

1.0

7.1944999695	0.0000000000	0.0000000000

0.0000000000	4.3975000381	0.0000000000

0.0000000000	0.0000000000	17.1418991089

Ca	P

12	8

Direct

0.511099994	0.000000000	0.157529995

0.011100000	0.000000000	0.842469990

0.988900006	0.000000000	0.157529995

0.488900006	0.000000000	0.842469990

0.511099994	0.500000000	0.657530010

0.011100000	0.500000000	0.342469990

0.988900006	0.500000000	0.657530010

0.488900006	0.500000000	0.342469990

0.500000000	0.000000000	0.500000000

0.000000000	0.000000000	0.500000000

0.500000000	0.500000000	0.000000000

0.000000000	0.500000000	0.000000000

0.250000000	0.000000000	0.313180000

0.750000000	0.000000000	0.686819971

0.250000000	0.500000000	0.813180029

0.750000000	0.500000000	0.186820000

0.750000000	0.000000000	0.961269975

0.250000000	0.000000000	0.038729999

0.750000000	0.500000000	0.461270005

0.250000000	0.500000000	0.538730025

**(b)**

Ca3P2_polar

1.0

7.1944918633	0.0000000000	0.0000000000

0.0000000000	4.3974761963	0.0000000000

0.0000000000	0.0000000000	17.1418933868

Ca	P

12	8

Direct

0.464411139	0.000000000	0.188324094

0.964411139	0.000000000	0.811675906

0.464411139	0.500000000	0.688324094

0.964411139	0.500000000	0.311675906

0.942214489	0.000000000	0.126729563

0.442214489	0.000000000	0.873270452

0.942214489	0.500000000	0.626729548

0.442214489	0.500000000	0.373270452

0.569694281	0.000000000	0.529996097

0.069694303	0.000000000	0.470003873

0.569694281	0.500000000	0.029996119

0.069694303	0.500000000	0.970003903

0.218458280	0.000000000	0.313178957

0.718458295	0.000000000	0.686821043

0.218458280	0.500000000	0.813178957

0.718458295	0.500000000	0.186821029

0.791401029	0.000000000	0.961270928

0.291401029	0.000000000	0.038729090

0.791401029	0.500000000	0.461270899

0.291401029	0.500000000	0.538729072

**(c)**

LiCdP_nonpolar

1.0

4.2581000328	0.0000000000	0.0000000000

-2.1290500164	3.6876228003	0.0000000000

0.0000000000	0.0000000000	7.3698000908

Li	Cd	P

2	2	2

Direct

0.000000000	0.000000000	0.500000000

0.000000000	0.000000000	0.000000000

0.333333343	0.666666687	0.750000000

0.666666687	0.333333343	0.250000000

0.333333343	0.666666687	0.250000000

0.666666687	0.333333343	0.750000000

**(d)**

LiCdP_polar

1.0

4.2688736916	0.0000000000	0.0000000000

-2.1344368458	3.6969530624	0.0000000000

0.0000000000	0.0000000000	7.3237400055

Li	Cd	P

2	2	2

Direct

0.000000000	0.000000000	0.999814034

0.000000000	0.000000000	0.499745488

0.666666687	0.333333343	0.233148426

0.333333343	0.666666687	0.733155787

0.666666687	0.333333343	0.592428923

0.333333343	0.666666687	0.092441313

Figure S2. Plain-text snapshots of VASP POSCAR files for (a) nonpolar Ca_3_P_2_, (b) polar Ca_3_P_2_, (c) nonpolar LiCdP, and (d) polar LiCdP.

Figure S3. Frequency-dependent bulk photovoltaic (shift current) responses ($\boldsymbol{\sigma}$) as a function of photon energy for (a) Ca_3_P_2_ and (b) LiCdP. Only the three tensor components exhibiting the largest magnitudes are presented for clarity; the remaining components are omitted as they are either negligible or identically zero within the considered photon energy range.

Figure S4. DFT-optimized crystal structures (a, b) and lattice parameters (c, d) of Al_0.75_Sc_0.25_N in the (a,c) nonpolar and (b,d) polar structures, respectively.

**Comparison between LiCdP and Al_0.75_Sc_0.25_N in PE curves**

We explain conventional methods to obtain an intrinsic coercive field from double-well potentials. The intrinsic coercive field can be estimated by solving the stationarity condition (δU/δP = 0) of the LGD free-energy functional or by numerically integrating the time-dependent Ginzburg–Landau (TDGL) equation until steady-state is reached, under an applied electric field.^[2,3]^

At first, we can write the energy functional as Eq (1).

$$U\left( P,\nabla P,E_{app} \right)$$

$$=\int_{V} \left[ \alpha P^{2}+\beta P^{4}+\gamma P^{6}+\delta P^{8}-\frac{\epsilon_{0}\epsilon_{r}}{2}E_{app}^{2} -E_{app} P+ \frac{G}{2}\left| \nabla P \right|^{2} \right]drdz$$

(1)

where 𝑈,𝑃, and 𝐸_app_ denote the energy density functional, polarization, and applied electric field, respectively. $\alpha, \beta, \gamma,$ and $\delta$ are the LGD expansion coefficients and 𝐺 is the gradient coefficient. The term $-\frac{\epsilon_{0}\epsilon_{r}}{2}E_{app}^{2}$ represents a background dielectric contribution; for a fixed applied field it is independent of 𝑃 and therefore does not affect the stationarity condition, but it can be retained when reporting the total energy density.

To describe polarization evolution toward local minima, we additionally employ the time-dependent Ginzburg–Landau (TDGL) dynamics, denoted as Eq (2).

$$\rho\frac{\partial P}{\partial t}=-\frac{\delta U\left( P,\left| \nabla P \right| \right)}{\delta P}=-2\alpha P-4\beta P^{3}-6\gamma P^{5}-8\delta P^{7}+E_{app}-G \nabla^{2}P$$

(2)

where 𝜌 is a kinetic coefficient. Solving the stationary condition (or, equivalently, evolving TDGL to steady state) provides the theoretical polarization response P(E_app_), from which the intrinsic hysteresis and coercive field can be extracted.

**Figure S5.** Intrinsic double-well potential and LGD coercive field analysis. **(a)** Calculated Landau-type energy density as a function of polarization for Al_0.75_Sc_0.25_N and LiCdP, showing characteristic double-well potentials. **(c)** Theoretical intrinsic P–E hysteresis loops calculated by minimizing the LGD free-energy functional using TDGL dynamics. The estimated intrinsic coercive field is ~32.5 MV/cm for Al_0.75_Sc_0.25_N and ~4.16 MV/cm for LiCdP, both of which exceed experimental values due to the absence of nucleation and domain-wall propagation effects in the homogeneous model.

Table S5. Summary table listing LGD expansion coefficients (α, β, γ, δ) fitted to the energy density curves in (a), along with the gradient energy coefficient G derived from the domain-wall energy (DWE) and the dielectric constant ($\boldsymbol{\epsilon}_{\boldsymbol{r}}$).

|  | Al_0.75_Sc_0.25_N | LiCdP |
| --- | --- | --- |
| $\alpha$ (J m/C^2^) | -4.46$\times$10^9^ | -4.09$\times$10^8^ |
| $\beta$ (J m^5^/C^4^) | 8.93$\times$10^9^ | 2.72$\times$10^8^ |
| $\gamma$ (J m^9^/C^6^) | -7.54$\times$10^9^ | -1.56$\times$10^8^ |
| $\delta$ (J m^13^/C^2^) | 2.06$\times$10^9^ | 3.65$\times$10^7^ |
| $\epsilon_{r}$ | 11.86 | 23.54 |
| $G$ (J m/C^2^) | 8.92$\times$10^9^ | 7.11$\times$10^7^ |

Based on these methods, we calculated the intrinsic coercive fields for LiCdP and Al_0.75_Sc_0.25_N. The polarization–energy-density relationship underlying the double-well potential (*U*(*P*)) is summarized in Figure S5a, and the corresponding LGD coefficients (fitted from the energy density profile and combined with the domain-wall energy) are reported as Table S5 (the domain-wall energy is 0.156 J m^-2^ for Al_0.75_Sc_0.25_N and 0.252 J m^-2^ for LiCdP).

As discussed in the main text (Figure 5), the energy difference between the paraelectric-like P=0 state and the ferroelectric state extracted from the calculated double-well potential is 0.138 eV/f.u. for LiCdP and 0.256 eV/f.u. for Al_0.75_Sc_0.25_N, i.e., roughly a factor of two. However, once the different unit-cell volumes are accounted for, the corresponding energy density (J m^−3^) differs more strongly, as shown in Figure S5a, leading to a substantially higher intrinsic coercive field for Al_0.75_Sc_0.25_N.

The intrinsic P–E curves obtained from the LGD description are summarized in Figure S5b. The intrinsic coercive field estimated for Al_0.75_Sc_0.25_N is approximately 32.5 MV cm^−1^ , which is about one order of magnitude larger than the experimentally reported value of roughly 4-7 MV cm^-1^.^[4–6]^

Such overestimation is widely recognized that intrinsic coercive fields derived from homogeneous LGD theory typically exceed experimental coercive fields because realistic switching proceeds through nucleation and domain-wall motion, processes not fully captured in the idealized intrinsic picture.^[2]^ Under the working assumption that a similar intrinsic-to-extrinsic reduction factor applies to LiCdP, the intrinsic estimate of ~4.16 MV cm^−1^ for LiCdP would correspond to an experimentally accessible coercive field on the order of ~500-900 kV cm^−1^, comparable to values in conventional ferroelectrics.^[7–13]^

Overall, these results suggest that LiCdP can achieve switching at substantially lower operating fields while maintaining a polarization magnitude comparable to Al_0.75_Sc_0.25_N. This combination—large polarization with reduced practical coercive field—highlights LiCdP as a promising candidate for low-power ferroelectric switching devices.

Table S6. Reaction pathways toward LiCdP evaluated using ARROWS^3^.^[14]^ Reaction energies ($\boldsymbol{\Delta}\boldsymbol{E}_{\boldsymbol{rxn}}$) are reported in meV per atom.

| Precursors | Reactions | $\Delta E_{rxn}$ |
| --- | --- | --- |
| Li + Cd + P | Li + Cd + P → LiCdP | −59.84 |
| Li_3_P + Cd + P | Li_3_P + Cd + P → LiCdP | 223.70 |
| Li + CdO + P | Li + CdO + P → LiCdP + O_2_ | 497.19 |
| Li_2_O + Cd + P | Li_2_O + Cd + P → LiCdP + O_2_ | 732.83 |
| Li + Cd + P_2_O­­_5_ | Li + Cd + P_2_O­_5_ → LiCdP + O₂ | 1294.87 |

**Feature-space positioning**

Figure S6 depicts the feature space of all generated structures onto a low-dimensional representation to contextualize the structural uniqueness of the identified ferroelectric candidates. The reference dataset used for training the MatterGen diffusion model was the Alex-MP20 database,^[15,16]^ which provides a diverse and chemically realistic baseline for crystalline materials. Structural features were computed using the CrystalNNFingerprint descriptor from Matminer,^[17]^ capturing the local coordination environment around each atom. These fingerprints were then embedded into a two-dimensional manifold using Uniform Manifold Approximation and Projection (UMAP), enabling a visual comparison of generated structures with known crystalline phases.^[18]^

Figure S6. UMAP representation of structural feature space. Structures from the Alex-MP-20 database are shown in light gray, generated candidates by the diffusion model in light blue, polar structures in dark blue, and the two final promising candidates as orange stars.

In Figure S6, the feature map shows a broad coverage of structural diversity. The Alex-MP20 structures populate the background space while the diffusion-generated structures occupy nearby regions confirming that the generative process faithfully reproduces realistic local environments. Within this space, polar structures are distributed with the screened subset. Moreover, the distribution reveals that two promising candidates do not form isolated outliers, but rather reside within the dense interior of the structural manifold indicating their chemical plausibility as they emerge naturally within the continuum of known structural chemistry rather than at its fringes.

To complement this feature-space visualization, we quantitatively evaluated the diversity and novelty of the generated structures using the Stability–Uniqueness–Novelty (S/U/N) metrics implemented in the MatterGen framework. This analysis confirms that all generated structures are structurally distinct from one another, yielding a uniqueness score of 100%, and that none correspond to known materials in the MP2020 reference dataset, resulting in a novelty score of 100%. Thermodynamic stability was explicitly enforced at a later stage through energy-above-hull filtering.

Particularly, the Ca_3_P_2_ and LiCdP appear within the core of the feature distribution. Their position inside the manifold rather than on its boundaries showing their compatibility with established crystallographic motifs and highlights the stability of their structural features. This embedding further demonstrates that generative design guided by diffusion models and validated through physical descriptors can yield materials that are not only novel but also structurally stable within the broader chemical space.

Figure S7. (a) Relative energies and (b) normalized volumes of the Ca_3_P_2_ structures along the MD trajectories at (c) 0 ps and (d) 100 ps during the simulations.

Figure S8. (a) Relative energies and (b) normalized volumes of the LiCdP structures along the MD trajectories at (c) 0 ps and (d) 100 ps during the simulations.

Figure S9. Temperature-dependent Helmholtz free energies of (a) Ca_3_P_2_ and (b) LiCdP.

**References**

[1]	N. N. Greenwood, A. Earnshaw, *Chemistry of the elements*, 2nd ed., Butterworth-Heinemann, Oxford Boston **1997**.

[2]	S. Ducharme, V. M. Fridkin, A. V. Bune, S. P. Palto, L. M. Blinov, N. N. Petukhova, S. G. Yudin, *Phys. Rev. Lett.* **2000**, *84*, 175.

[3]	T. Rollo, F. Blanchini, G. Giordano, R. Specogna, D. Esseni, *Nanoscale* **2020**, *12*, 6121.

[4]	Md. R. Islam, N. Wolff, M. Yassine, G. Schönweger, B. Christian, H. Kohlstedt, O. Ambacher, F. Lofink, L. Kienle, S. Fichtner, *Applied Physics Letters* **2021**, *118*, 232905.

[5]	P. Wang, D. Wang, N. M. Vu, T. Chiang, J. T. Heron, Z. Mi, *Applied Physics Letters* **2021**, *118*, 223504.

[6]	D. Wang, J. Zheng, P. Musavigharavi, W. Zhu, A. C. Foucher, S. E. Trolier-McKinstry, E. A. Stach, R. H. Olsson, *IEEE Electron Device Lett.* **2020**, *41*, 1774.

[7]	A. I. Mardare, C. C. Mardare, E. Joanni, *Journal of the European Ceramic Society* **2005**, *25*, 735.

[8]	G. L. Rhun, G. Poullain, R. Bouregba, G. Leclerc, *Journal of the European Ceramic Society* **2005**, *25*, 2281.

[9]	A. Datta, D. Mukherjee, S. Witanachchi, P. Mukherjee, *Adv Funct Materials* **2014**, *24*, 2638.

[10]	D. H. Li, E. S. Lee, H. W. Chung, S. Y. Lee, *Applied Surface Science* **2006**, *252*, 4541.

[11]	K. R. Udayakumar, P. J. Schuele, J. Chen, S. B. Krupanidhi, L. E. Cross, *Journal of Applied Physics* **1995**, *77*, 3981.

[12]	S. Samanta, V. Sankaranarayanan, K. Sethupathi, *Vacuum* **2018**, *156*, 456.

[13]	E. M. Lee, Y. Ahn, J. Y. Son, *Current Applied Physics* **2020**, *20*, 755.

[14]	N. J. Szymanski, P. Nevatia, C. J. Bartel, Y. Zeng, G. Ceder, *Nat Commun* **2023**, *14*, 6956.

[15]	A. Jain, S. P. Ong, G. Hautier, W. Chen, W. D. Richards, S. Dacek, S. Cholia, D. Gunter, D. Skinner, G. Ceder, K. A. Persson, *APL Materials* **2013**, *1*, 011002.

[16]	J. Schmidt, H.-C. Wang, T. F. T. Cerqueira, S. Botti, M. A. L. Marques, *Sci Data* **2022**, *9*, 64.

[17]	L. Ward, A. Dunn, A. Faghaninia, N. E. R. Zimmermann, S. Bajaj, Q. Wang, J. Montoya, J. Chen, K. Bystrom, M. Dylla, K. Chard, M. Asta, K. A. Persson, G. J. Snyder, I. Foster, A. Jain, *Computational Materials Science* **2018**, *152*, 60.

[18]	L. McInnes, J. Healy, J. Melville, *UMAP: Uniform Manifold Approximation and Projection for Dimension Reduction*, arXiv **2018**.
